# Effect of Low-Intensity Pulsed Ultrasound (LIPUS) on Alveolar Bone during Maxillary Expansion Using Clear Aligners

**DOI:** 10.1155/2022/4505063

**Published:** 2022-04-29

**Authors:** Maha Bahammam, Tarek El-Bialy

**Affiliations:** ^1^Periodontics, Department of Periodontology, Faculty of Dentistry, King Abdulaziz University, Jeddah, Saudi Arabia; ^2^Executive Presidency of Academic Affairs, Saudi Commission for Health Specialties, Riyadh 11614, Saudi Arabia; ^3^Orthodontics and Biomedical Engineering, Division of Orthodontics, Katz Group Centre for Pharmacy and Health Research, School of Dentistry, University of Alberta, Edmonton, AB, Canada T6G 1C9

## Abstract

The present study evaluated the possible effect of low-intensity pulsed ultrasound (LIPUS) on buccal bone plate thickness and height after maxillary arch expansion using clear aligners. The cone beam computed tomography (CBCT) of before and immediately after maxillary arch expansion (3 mm per side) of 28 adult patients (18 in LIPUS group and 10 in control) (average age 36.2 ± 13.2 years old) was analyzed. The wearing protocol of clear aligners in the LIPUS group was to change the aligners every 4 to 5 days, while the wearing protocol in the control group (without LIPUS) was to change the aligners every 7 to 10 days. Bone thickness at 3 mm and 6 mm from the buccal alveolar bone crests, along with the measurements of buccal alveolar bone heights, was measured in standardized sagittal sections. Data were analyzed through paired sample *t*-test and the Wilcoxon test. The results were given as mean ± standard deviation and 95% confidence intervals. *p* value < 0.05 was considered statistically significant. The results showed significant increase in bone height in both groups. However, comparison of both LIPUS and control groups showed no statistically significant difference in bone thickness or bone height. The results of this study showed that the use of LIPUS together with accelerated aligner tray change protocol did not affect alveolar bone integrity when compared to the control group.

## 1. Introduction

Dental crowding can be alleviated without extractions, and hence, the extraction therapy may be avoided. Interproximal reduction (IPR) is one of the methods that may be utilized during orthodontic treatment to create extra intra-arch space in patients with mild to moderate crowding [1], but sagittal extension beyond the skeletal base limits may increase the risk of bone dehiscence and retraction of gingival margin from the crown of teeth [2–4]. Transverse expansion can be accomplished by releasing the external muscle pressure to promote the subperiosteal bone formation [5], utilizing buccal pressure with palatal devices, and more recently using fixed appliances with wide arch wires [6, 7].

According to three-dimensional (3D) studies [8], rapid palatal expansion causes horizontal and vertical reductions in the buccal alveolar bone of premolars and molars, whereas the combination of self-ligating brackets and heat-activated super elastic arch wires is said to produce a low-force, low-friction environment in which the bone follows tooth movement. As a result, orthodontic therapy that uses self-ligating equipment would allow for more dental expansion, less incisor proclination, and fewer extractions than traditional orthodontic treatment [9].

CBCT studies [3, 10] in nonextraction patients examining the bone at the maxillary second premolars and mandibular incisors have failed to show that self-ligating appliances promote expansion with torque control and those teeth are followed by the alveolar bone. There is yet to be published more thorough research examining alterations in the buccal bone in both the posterior and anterior areas of the maxilla.

A study conducted by Maurya et al. [11] reported that the rate of tooth movement increases through intermittent use of LIPUS that was extra orally applied by the operator to one side of the mouth between 3 and 15 days. However, a recent study on daily use of LIPUS showed accelerated tooth movement and a reduction of orthodontic treatment time between 29 and 50% [12]. LIPUS has been shown to enhance fracture healing and bone regeneration or periodontal reconstruction [13]. It is also proven to improve bone remodeling, maturation, quality, and volume of the alveolar bone [13]. Considering its numerous benefits, it is important to study its effect on alveolar bone during maxillary arch expansion using clear aligners for better decision-making during orthodontic treatment.

There is no report on the effect of LIPUS on bone integrity during dental arch expansion. Therefore, the aim of this study is to examine the effect of LIPUS with accelerated aligner change on alveolar bone thickness (BT) and bone height (BH) during maxillary arch expansion using clear aligners. Our null hypothesis was that no difference exists in bone thickness or bone heights after maxillary arch expansion with or without the use of LIPUS with accelerated aligner change. The alternative hypothesis was that LIPUS with accelerated aligner change increases bone thickness and height after maxillary arch expansion.

## 2. Material and Methods

The health ethics committee at the University of Alberta approved this retrospective study (protocol number Pro00091339). The records of 28 patients (average ages 36.2 ± 13.2 years old) treated by either Invisalign SmartTrack® clear aligners only (*N* = 10) or in combination with LIPUS (*N* = 18) from December 2017 to April 2019 were evaluated.

The wearing protocol of clear aligners in the control group (without LIPUS) was to change the aligners every 7 to 10 days, while the wearing protocol in the LIPUS group was to change the aligners every 4 to 5 days.

All the patients included in this study had a maxillary arch expansion of 3 mm as well as had before and immediately after treatment CBCTs as part of their regular orthodontic treatment records. Since the introduction of CBCT technology, significant advancements in hardware and software components have lowered the patient's radiation dosage. Changes in sensor technology, a narrower field of vision depending on the application, and pulsed radiation techniques that follow ALARA's radiation dose concept of “as low as reasonably achievable” are among these enhancements [14]. The small and unbalanced sample size was obtained as there were few cases with the posttreatment radiographs. CBCT settings are presented in Table 1.

CBCT images were acquired using ICAT (Imaging Sciences International (ISI), PA, USA). It was used to scan the angular and linear evaluations. The correct alignments of the samples were obtained through scout view in the scanner according to the adjusted light beam before obtaining the intended field view. The instrument was supplied with an amorphous silicon flat panel sensor with cesium iodide (CsI) scintillator, 0.123 mm image resolution, 0.5 mm focal spot size, and 14 bit grayscale resolution.

The data were exported and transferred in Digital Imaging and Communications in Medicine (DICOM) format after the acquisition and downloaded to a personal computer via a compact disk (CD) for analysis. The computer had OnDemand3D Application software (Cybermed, South Korea) to obtain linear and angular measurements. Moreover, the 3D Ceph module was used for fulfilling the given tasks. Superimposition of pretreatment (T0) and posttreatment (T1) scans was performed to identify landmarks similarly between sequential scans and apply corrective positioning points to align both scans at the same orientation during measurement because all the patients were not positioned in the same direction. This helped to standardize the position of patients' datasets between pretreatment and posttreatment scans while ensuring a perfect fit between both scans.

**Figure 1:**
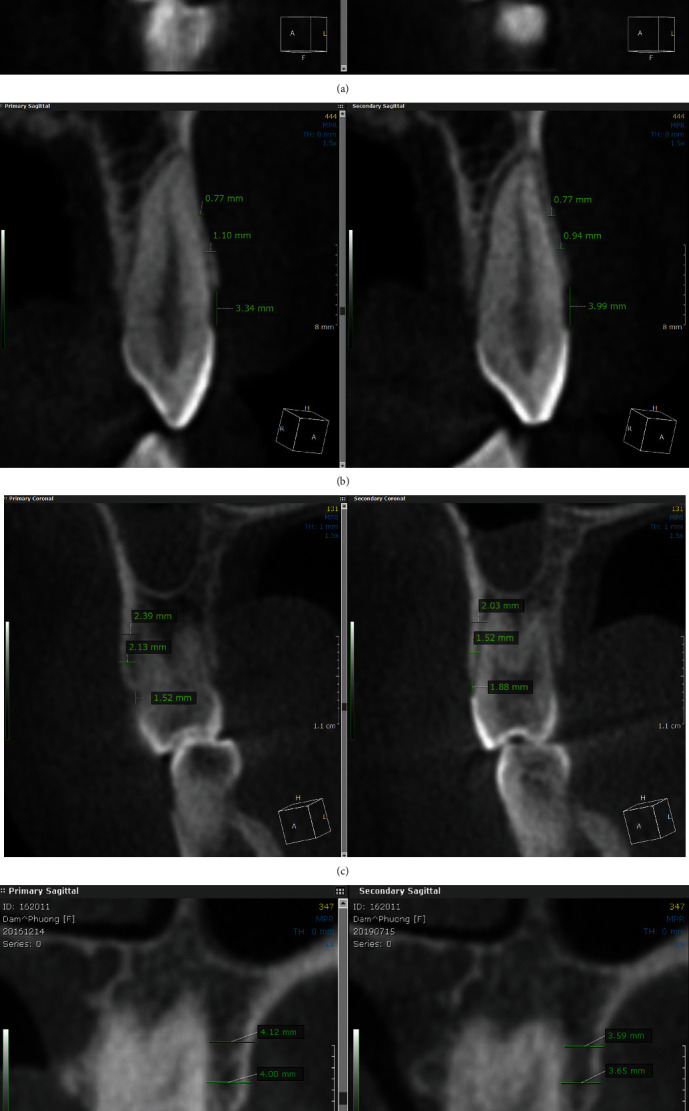
Obtaining measurements and recordings. Second premolar of the control group (a) before treatment (primary sagittal) and (b) after treatment (secondary sagittal). Procedure to obtain accurate measurements. (c) Bone thickness at the canine area before (left) and after (right) treatment with clear aligner only (control group). (d) Bone thickness at the canine area before (left) and after (right) treatment with LIPUS with clear aligner (experimental group).

The fusion module in OnDemand3D was utilized for completely automated voxel-based rigid registration. The scans were aligned and positioned in a way to examine the tooth at pre- and posttreatment scans at the same time. The sagittal, axial, and coronal planes were oriented to coincide with the long axis of the tooth. In the next step, the coronal plane was assigned to acquire the measurements. The levels of the cementoenamel junction at the buccal surface and the lowest point of the alveolar crest were identified. Later, simultaneous measurements and recordings were obtained for the global distance between the two points at the same cut, but two different scans (Figure 1(a)). A three-millimeter line was drawn parallel to the buccal plate for measuring the thickness of alveolar bone, starting from the lowest point of the alveolar crest. This was followed by another three-millimeter line upwards as well to identify the 3 mm and 6 mm levels from the alveolar crest level. At the end of these two lines, horizontal measurement was taken from the outer surface of the buccal bone to the periodontal membrane space inwards of the associated tooth (Figure 1(b)). This was repeated at both the scans for a single tooth.

For calibration, 10 CBCT images were randomly selected independent of the time interval, and all measurements were performed two times by the same operator within a 2-week interval. All evaluated measurements had an intraclass correlation coefficient (ICC) greater than 93%, indicating the reliability of the measurements at both assessment times. According to a preliminary power analysis, a minimum sample size of 28 participants was calculated to achieve an 80 percent power of the study at a significance level of .05, to demonstrate true difference of 2.5 mm^2^ in the buccal bone area of the second premolar, assuming a previously reported standard deviation of 3.6 mm [12].

### 2.1. LIPUS Application

Group 1 (LIPUS group) consisted of 18 patients who received LIPUS therapy and clear aligners therapy. The LIPUS group patients were instructed to change the aligners every 4 to 5 days. LIPUS was administered for 20 minutes every day while utilizing anultrasound instrument. The gadget featured a mouthpiece that looked like a mouthguard that was linked to the portable electronics, which had a screen that displayed treatment information. The transducers are inserted in the mouthpiece, which was placed at the level of the tooth root. Coupling gel was placed on the inside of the mouthpiece before each treatment to ensure adequate LIPUS transmission from the mouthpiece through the gums to the dental roots. Ultrasound at a frequency of 1.5 MHz, a pulse repetition rate of 1 KHz, and average output intensity of 30 mW/cm^2^ was used in the LIPUS output.

Group 2 (the control group) consisted of 10 patients who received no LIPUS therapy. These patients were treated with clear aligners therapy only and were instructed to change the aligners every 7 to 10 days.

Data were analyzed using SPSS version 20. The normality of the distribution of the variables was assessed by the Shapiro-Wilk test. Interphase changes (T1–T0) were calculated, and if normally distributed, these were compared using paired *t*-tests; if this was not the case, the Wilcoxon test was used. All pretreatment and posttreatment radiographs were read by professional radiologists. Paired sample *t*-test was used for identifying the differences between bone thickness (BT) at 3 mm (BT-3) and 6 mm (BT-6) for clear aligner and LIPUS and for comparing mean differences between the clear aligner and LIPUS with clear aligner groups for bone height (BH) and BT-3, respectively. The results were given as mean ± standard deviation and 95% confidence intervals. *p* value < 0.05 was considered statistically significant. The intraexaminer reliability assessment was achieved through statistical analysis using SPSS and 95% confidence interval for ICC values. The reliability ranking was described using ICC values (<0.9 = excellent, 0.9–0.76 = good, 0.75–0.5 = moderate, and >0.5 = poor) [15].

## 3. Results

The statistical analyses showed that few measurements were statistically significant (T1-T0).  Table 2 further provides the difference in bone height (control) and LIPUS before and after treatment with a clear aligner. There was statistically significant increase in bone height at the right second molar (*p* value = 0.017), right first molar (*p* value = 0.032), right second premolar (*p* value = 0.01), right canine (*p* value = 0.036), left second molar (*p* value = ≤0.0010), and left first molar (*p* value = 0.052) after using clear aligners. In addition, a statistically significant increase in bone height of right second molar (*p* value = 0.03), right first molar (*p* value = 0.05), and left second molar (*p* value = 0.01) was observed after applying LIPUS with clear aligners (Table 2).

Table 3 shows the statistically significant decrease in alveolar bone thickness at 3 mm from the cementoenamel junction of the right second molar (*p* 0.040), left second molar (*p* 0.033), and left second premolar (*p* 0.035) after applying clear aligners. Similarly, a statistically significant decrease in alveolar bone width at 3 mm was obtained for all the tested molars and premolars after applying LIPUS with clear aligners (*p* < 0.05).

Table 3 provides the comparative analysis of bone thickness at 6 mm level between clear alignment (control) and LIPUS with a clear aligner. Findings indicated no significant difference in alveolar bone thickness at 6 mm apical from the cementoenamel junction at any of the molars and premolars in the control group, whereas applying LIPUS with clear aligners showed significant decrease at alveolar bone width at 6 mm apical from the cementoenamel junction for right second molar (*p* ≤ 0.0010), right second premolar (*p* 0.03), right first premolar (*p* 0.02), right first premolar (*p* 0.03), right canine (*p* 0.05), left second molar (*p* value = ≤0.0010), left second premolar (*p* 0.02), and left first premolar (*p* 0.03).

Table 4 shows a comparison of bone height, BT-3, and BT-6 between clear aligner (control) and LIPUS with clear aligner groups. The results demonstrated no significant difference in bone height between both groups (*p* > 0.05). BT-3 analysis illustrated a significant decrease in alveolar bone width at 3 mm from the cementoenamel junction between both groups in two teeth (right first premolar (*p* 0.051) and left first molar (*p* 0.035)), while BT-6 results illustrated no significant decrease in alveolar bone thickness at 6 mm from the cementoenamel junction between control and LIPUS groups (*p* > 0.05).

The bone thickness was observed before and after using a clear aligner (control) and LIPUS with a clear aligner (experimental) at the canines, respectively, and illustrated in Figures 1(c) and 1(d).

## 4. Discussion

The present study attempted to test the effect of LIPUS on bone thickness after maxillary arch expansion using clear aligners. The findings of this study supported the null hypothesis that no difference exists in bone thickness or bone heights after maxillary arch expansion with or without the use of LIPUS with accelerated tray change. There are many possible explanations for the present study results. One explanation could be that during orthodontic tooth movement, the application of LIPUS with accelerated tray change increases bone resorption more than bone deposition. This may be in agreement with previous research that showed that LIPUS enhances osteoclastic activities in vitro [16], ex vivo, and in animals [17, 18]. The immediate CBCT after active orthodontic treatment may have not provided the possible anabolic effect of LIPUS on bone thickness and heights during the retention period (without orthodontic tooth movement).

The main difference between results reported by El-Bialy et al. [12] regarding the effect of LIPUS on tooth movement could be due to the difference between the two groups in terms of the rate of LIPUS application. Maurya et al.'s group [11] applied LIPUS between 3- and 15-day intervals compared to El-Bialy et al.'s group [12, 17] that used LIPUS on daily basis. This difference is in agreement with former studies that showed that the LIPUS effect is dose (applications rate) dependent [13, 19].

Although the clear aligners were changed faster in the LIPUS group (every 4 to 5 days) as compared to the control group (every 7 to 10 days), no fenestration or dehiscence was noted with LIPUS-treated teeth/group. This could be explained by the fact that although bone remodeling increased with LIPUS, there might be the preservation of the buccal periosteum at the same time by LIPUS which might have prevented fenestration or dehiscence formation during orthodontic treatment. This might be better confirmed by future animal experiments. It is to be noted that previous research has shown that LIPUS increases osteoblastic activity as well as periodontal cells [20–22].

The thickness of the anterior alveolus is known as a limiting factor in any orthodontic treatment [23]. The risk of treatment-related alveolar defects and bone loss increases because of the thickened anterior alveolus that acts as an anatomical boundary. Alveolar defects are not solely considered as orthodontic tooth movement because they can be detected easily among the untreated patients. The present study showed no statistically significant difference in bone thickness between the LIPUS group with accelerated tray change and the control.

One of the previous studies has reported an increase in buccal bone thickness after several months of the retention period after maxillary rapid expansion [16]. The accuracy of tooth movement using clear aligners in integrated three-dimensional digital models was investigated by Zhang et al. [24] who showed that clear aligners can shift crowns but not roots of anterior teeth to desired locations because they promote tooth movement through a tipping motion, while displacement differences for roots on an integrated model after treatment with fixed appliances were assessed by Lee et al. [25]. The results of the above-discussed studies showed that accurate visualization of 3-dimensional positions of all teeth is provided by an integrated model with 3-dimensional crowns and roots, with no additional radiation. Similarly, relationships between roots and alveolar bone can be observed using the integrated model with 3-dimensional positions of the jaws; however, it fails to provide accuracy and reliability.

The retention phase of orthodontic treatment that holds the teeth in the corrected position after the completion of orthodontic therapy is an important phase of teeth stability for the treatment success. Moreover, recent studies also suggested moving teeth through distraction techniques into the regenerated alveolar bone [26]. However, the time for tooth movement is controversial. Some studies suggest that it should be done after 2-3 months, as consolidation in regenerated bone requires sufficient time [27]. Another technique of noninvasive stereophotogrammetry generate 3-dimensional surface model with clinical accuracy in short capture time and use successfully for face scanning. Laser is in use as an alternative facial scanner to stereophotogrammetry to reduce facial prostheses and note facial measurements [28]. However, Lucchese et al. [29] suggested that vigilant assessment should be done on patient's undertaking distraction osteogenesis, and then, the ideal time for tooth movement should be evaluated to decrease the treatment time in the orthodontic phase of postdistraction.

Janson et al. [30] studied and compared the alveolar bone crest heights among patients treated with either bioefficient therapy, the edgewise straight wire system, or the simplified standard edgewise technique. All the treated groups showed a significant difference in cementoenamel junction to alveolar bone distance, while rapid maxillary arch expansion has been reported to cause buccal displacement of anchor teeth. However, Digregorio et al. [31] reported that rapid maxillary arch expansion does not decrease the thickness of the buccal bone plate of the maxillary 1st molars when the mixed dentition with the appliance is anchored to deciduous teeth, whereas when permanent dentition is used as anchors, it reduces the thickness of the buccal bone plate. Another study [32] examined maxillary buccal bone changes during orthodontic expansion first year with clear aligner (Invisalign), and no substantial changes in bone measurements were reported.

Patients with rising numbers showing active signs of TMJ engagement can be safely treated with RME for at least one year, anticipating similar advantages to those of healthy patients [33]. Rheumatologists and dentists must be informed of the safety and possible advantages of palatal expansion in patients for enhancing the outcome of orthodontic treatment and mitigating the presence of more invasive procedures [33]. Lastly, most emergencies can be managed by teleorthodontists to reassure and follow patients remotely. The objective predefined by teleassistance was fulfilled as it mitigated office visits of patients while retaining regular monitoring regardless of compromising the outcomes.

## 5. Limitations

The primary limitation of the study is a small sample size and an unequal number of patients in both groups, and follow-up CBCT was not performed except the immediate CBCT after treatment. Another limitation of the study was that bone height and thickness depend on the amount of root movement. Therefore, crown and root apex expansion should be measured to understand the type of tooth movement performed, which was not done in this study. Moreover, the age of the patients in this study had a large range. There could have been some differences in the effect of treatment among teenagers and adults. Furthermore, due to inherently being a retrospective study, it was limited in conducting a method error study.

## 6. Conclusion

The immediate CBCT after maxillary arch expansion using clear aligners' treatment showed that the use of LIPUS with accelerated tray change protocol did not affect alveolar bone integrity compared to the control group. Future prospective studies are suggested to check the outcomes with a larger sample size, controlled/matched age groups, and the method error study of the instrument.

## Figures and Tables

**Table 1:** Machine protocols.

Tube voltage	120 kVp
Milliampere	10 mA
Voxel size	0.3 mm
Scanning time	8.9 seconds
Field of view	13 cm height∗16 cm diameter
Rotation degree	360°

**Table 2:** Difference in bone height for clear aligner (control) and LIPUS with clear aligner.

Tooth number	Clear aligner (control)				LIPUS with clear aligner			
	BH before	BH after	Mean difference (SD)	*p* value	BH before	BH after	Mean difference (SD)	*p* value
Rt second molar	1.70	2.04	-0.34 (0.34)	0.017	1.48	1.80	0.22 (0.39)	0.03
Rt first molar	2.09	2.31	-0.23 (0.30)	0.032	2.14	1.62	-0.48 (0.98)	0.05
Rt second premolar	1.21	1.38	-0.2 (0.15)	0.01	1.41	1.36	0.03 (0.81)	0.88
Rt first premolar	2.46	2.84	-0.38 (0.67)	0.126	2.445	1.83	-0.61 (1.43)	.086
Rt canine	2.17	2.38	-0.21 (0.25)	0.036	1.79	1.56	-0.24 (1.03)	0.34
Lt second molar	1.93	2.01	0.08 (0.04)	≤≤0.0011	1.44	1.81	0.38 (0.52)	0.01
Lt first molar	2.04	2.46	-0.42 (0.55)	0.052	1.82	1.49	-0.32 (1.00)	0.19
Lt second premolar	1.40	1.44	-0.03 (0.17)	0.592	1.27	1.08	-0.19 (0.97)	0.41
Lt first premolar	2.37	2.97	-0.59 (2.02)	0.404	2.54	1.94	-0.59 (1.55)	0.12
Lt canine	2.71	2.65	0.06 (1.04)	0.864	1.99	1.91	-0.1 (0.76)	0.61

**(a):** 

Tooth number	Clear aligner (control)				LIPUS with clear aligner			
	BT-3 mm before	BT-3 mm after	Mean difference (SD)	*p* value	BT-3 mm before	BT-3 mm after	Mean difference (SD)	*p* value
Rt second molar	2.62	2.30	0.32 (0.39)	0.040	2.33	2.07	-0.25 (0.30)	≤0.001
Rt first molar	0.69	0.60	0.09 (0.14)	0.078	0.90	0.55	-0.33 (0.55)	0.02
Rt second premolar	1.62	1.47	0.14 (0.41)	0.321	1.40	0.88	-0.49 (0.46)	≤0.001
Rt first premolar	1.00	0.82	0.18 (0.32)	0.124	1.10	0.55	-0.56 (0.58)	≤0.001
Rt canine	0.56	0.52	0.04 (0.11)	0.347	0.67	0.38	-0.29 (0.47)	0.02
Lt second molar	2.19	1.96	0.23 (0.27)	0.033	2.10	1.90	-0.20 (0.27)	≤0.001
Lt first molar	1.03	0.69	0.34 (0.61)	0.132	0.92	0.53	-0.36 (0.50)	0.01
Lt second premolar	1.37	1.26	0.11 (0.13)	0.035	1.32	0.99	-0.33 (0.42)	≤0.001
Lt first premolar	0.39	0.27	0.11 (0.19)	0.111	0.98	0.61	-0.37 (0.67)	0.03
Lt canine	0.37	0.34	0.03 (0.10)	0.484	0.77	0.45	-0.32 (0.52)	0.03

**(b):** 

Tooth number	Clear aligner (control)				LIPUS with clear aligner			
	BT-6 mm before	BT-6 mm after	Mean difference (SD)	*p* value	BT-6 mm before	BT-6 mm after	Mean difference (SD)	*p* value
Rt second molar	1.74	1.57	0.16 (0.25)	0.090	2.73	2.51	-0.21 (0.29)	≤0.001
Rt first molar	0.63	0.47	0.17 (0.36)	0.203	0.84	0.63	-0.20 (0.49)	0.10
Rt second premolar	1.51	1.60	-0.1 (0.39)	0.479	1.09	0.64	-0.42 (0.75)	0.03
Rt first premolar	0.25	0.19	0.06 (0.12)	0.156	0.72	0.33	-0.39 (0.63)	0.02
Rt canine	0.19	0.15	0.03 (0.08)	0.222	0.41	0.21	-0.19 (0.39)	0.05
Lt second molar	2.19	2.00	0.19 (0.44)	0.246	2.53	2.33	-0.21 (0.27)	≤0.001
Lt first molar	0.86	0.66	0.20 (0.45)	0.215	0.80	0.54	-0.25 (0.51)	0.06
Lt second premolar	1.24	1.26	-0.03 (0.15)	0.607	0.93	0.77	-0.15 (0.26)	0.02
Lt first premolar	0.11	0.08	0.03 (0.09)	0.347	0.71	0.43	-0.28 (0.52)	0.03
Lt canine	0.24	0.21	0.03 (0.07)	0.173	0.44	0.33	-0.11 (0.31)	0.168

**Table 4:** Comparing BH, BT-3, and BT-6 between clear aligner (control) and LIPUS with clear aligner groups.

	Mean	Std. deviation	*t*	df	Sig. (2-tailed)
Rt second molar	-0.333	0.707	-1.414	8	0.195
Rt first molar	0.333	1.000	1.000	8	0.347
Rt second premolar	0.222	0.833	0.800	8	0.447
Rt first premolar	0.556	1.878	0.887	8	0.401
Rt canine	0.333	1.000	1.000	8	0.347
Lt second molar	-0.333	0.707	-1.414	8	0.195
Lt first molar	0.222	1.302	0.512	8	0.622
Lt second premolar	.444	1.424	0.936	8	0.377
Lt first premolar	1.000	3.041	0.986	8	0.353
Lt canine	0.524	1.325	1.118	7	0.301
Rt second molar	0.333	0.500	2.000	8	0.081
Rt first molar	0.222	0.667	1.000	8	0.347
Rt second premolar	0.556	0.882	1.890	8	0.095
Rt first premolar	0.556	0.726	2.294	8	0.051
Rt canine	0.111	0.333	1.000	8	0.347
Lt second molar	0.444	0.726	1.835	8	0.104
Lt first molar	0.889	1.054	2.530	8	0.035
Lt second premolar	0.333	0.500	2.000	8	0.081
Lt first premolar	0.556	1.014	1.644	8	0.139
Lt canine	0.250	0.463	1.528	7	0.170
Rt second molar	0.222	0.441	1.512	8	0.169
Rt first molar	0.222	.833	0.800	8	0.447
Rt second premolar	0.444	1.130	1.180	8	0.272
Rt first premolar	0.333	0.707	1.414	8	0.195
Rt canine	0.111	0.333	1.000	8	0.347
Lt second molar	0.222	0.441	1.512	8	0.169
Lt first molar	0.556	0.882	1.890	8	0.095
Lt second premolar	0.222	0.441	1.512	8	0.169
Lt first premolar	0.222	0.667	1.000	8	0.347
Lt canine	0.125	0.354	1.000	7	0.351

## Data Availability

The data of this study will be furnished upon reasonable request from the corresponding author.
